# Tomato SlRUP is a negative regulator of UV-B photomorphogenesis

**DOI:** 10.1186/s43897-021-00010-z

**Published:** 2021-08-27

**Authors:** Chunli Zhang, Qianwen Zhang, Hongye Guo, Xiaohui Yu, Wenjing Liang, Yinhua Chen, Ruohe Yin, Li Lin

**Affiliations:** 1 School of Agriculture and Biology, Shanghai Jiao Tong University, 800 Dongchuan RD, Minhang District, Shanghai, 200240 People’s Republic of China; 2 Key Laboratory of Urban Agriculture (Ministry of Agriculture) and Joint Center for Single Cell Biology, School of Agriculture and Biology, Shanghai Jiao Tong University, Shanghai, 200240 People’s Republic of China; 3 Hainan Key Laboratory for Sustainable Utilization of Tropical Bioresource, Hainan University, Haikou, 570228 People’s Republic of China

## Introduction

Sunlight is an environmental factor regulating plant growth and development. Plants have evolved a battery of photoreceptors to sense and respond to different wavelengths of light (Jenkins, 2017; Liu et al., 2020a). UV-B (280–315 nm) comprises the shortest wavelength of sunlight on the earth surface. In general, low fluence rate UV-B does not generate damage effects on plants, rather it induces plant photomorphogenic responses (Yin and Ulm, 2017). High fluence rate UV-B may damage macromolecules, including nucleic acids, protein and membranes (Hideg et al., 2013). The UV-B photoreceptor UVR8 protein ((UV RESISTANCE LOCUS 8) perceives the low dose UV-B and triggers UV-B specific signal transduction. The photomorphogenic responses mediated by UVR8 play a key role in UV-B acclimation and plant survival in nature (Favory et al., 2009). UVR8 exists as homodimer in its ground state and UV-B perception leads to dimer dissociation into monomer, which is the active form for signaling (Rizzini et al., 2011). In Arabidopsis, RUP1 (REPRESSORS OF UV PHOTOMORPHOGENESIS 1) and RUP2 are two key negative regulators of UV-B signaling (Gruber et al., 2010). RUP1 and RUP2 promote the reversion of UVR8 monomer to homodimer (Heijde and Ulm, 2013).

Tomato (*Solanum lycopersicum*) is an agriculturally and economically important vegetable crop. In agriculture, tomato seedlings may face sudden UV-B exposure when transferred from seedling bed in green house, where UV-B is blocked by plastic cover or glass, to open field. Therefore, it is important to investigate how tomato seedlings respond to UV-B. Previously, we demonstrated that Tomato UVR8 (SlUVR8) regulates seedling photomorphogenesis and UV-B stress tolerance (Liu et al., 2020b). However, the negative regulation of UV-B signaling is not known.

## Results

**Fig. 1:**
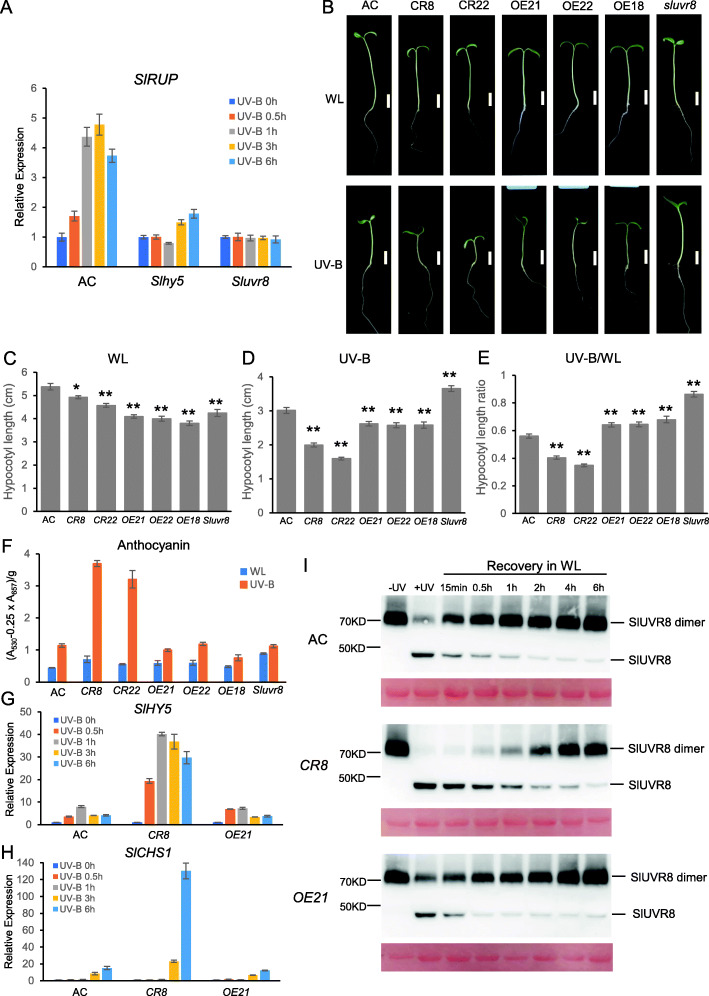
SlRUP negatively regulates the UVR8-mediated UV-B photomorphogenesis. (A) Transcript analysis of *SlRUP* in response to UV-B in *Sluvr8*, *Slhy5* mutants compared with wildtype (AC, *Ailsa Craig*) by qRT-PCR. 6d old seedlings were irradiated with UV-B for different lengths. Mean and SE of three biological replicates are presented. (B) Representative seedlings of wildtype and *Slrup* mutants (*CR8* and *CR22*), and *SlRUP-GFP* lines grown in white light or white light supplemented with UV-B. Bar = 1 cm. (C) and (D) Hypocotyl length of 6 d old seedlings grown under white light (WL) or white light supplemented with UV-B (UV-B), Mean and SE are presented (*n* = 30). (E) The hypocotyl length ratio of UV-B/WL. Asterisks indicate significant differences between transgenic lines and wildtype, differences with a *P* < 0.05 (*) and *P* < 0.01 (**) resulting from a one-way analysis of variance test. (F) UV-B induced anthocyanin accumulation. Mean and SE are presented (n = 3). (G) and (H) qRT-PCR analysis of *SlHY5* and *SlCHS1* in response to UV-B in wildtype, *Slrup* mutant, and *SlRUP-GFP* line for different UV-B radiation time. Mean and SE of three biological samples are presented. (I) SlRUP promotes redimeriation of SlUVR8 post UV-B treatment. 6 d old seedlings were irradiated with broadband UV-B for 0.5 h, followed by recovery in white light (WL) in the absence of UV-B for different time. Anti-SlUVR8 was used as the primary antibody for immunoblot analysis. Ponceau staining serves as loading control

In Arabidopsis, the two highly related WD40 proteins RUP1 and RUP2 (AtRUP1 and AtRUP2) were identified to be repressors of UVR8-mediated UV-B signaling pathway (Gruber et al., 2010). We found that AtRUP1 and AtRUP2 show high identity to only one putative protein encoded by *Solyc11g005190* in Tomato genome (55.5 and 56.1%, respectively). The *Solyc11g005190* was therefore called tomato *RUP (SlRUP).* Previously, SlRUP was shown to be a negative regulator of photomorphogenesis in white light, where it was named as LeCOP1Like (Liu et al., 2004). For clarity we renamed LeCOP1Like as SlRUP herein. Like AtRUP1 and AtRUP2, SlRUP mainly consists of WD40-repeats (Trp-Asp) domain with apparently no additional domains (Fig. S1A&B). Transcript of *SlRUP* was induced by UV-B in a SlUVR8- and SlHY5-dependent manner (Fig. 1A).

To analyze the physiological functions of SlRUP, we generated *SlRUP* overexpression lines with C-terminal GFP, and *Slrup* mutants by CRISPR/CAS9 approach (Fig. S2). Three independent *SlRUP-GFP* overexpression lines were selected by immunoblot analysis (Fig.S2A). Two homozygous *Slrup* mutant lines were identified by PCR-resequencing, with 111 bp and 113 bp deletion, respectively (Fig.S2B-E). Under white light, *Slrup* mutants developed shorter hypocotyls than wildtype (AC, *Ailsa Craig*) (Fig. 1B&C). UV-B can inhibit hypocotyl elongation of seedlings, the hypocotyl length of wildtype seedling under UV-B was only about 56% of that under white light (Fig. 1D&E). *Slrup* mutants and *RUP-GFP* overexpression seedlings were hypersensitive and hyposensitive to UV-B, respectively (Fig. 1B, D, E). Consistent with the key function of SlUVR8 in UV-B signaling, hypocotyl length of *Sluvr8* mutant was only slightly shortened under UV-B in comparison to that under white light. Relative to white light control, UV-B induced anthocyanin content to about 3 folds in wildtype, 6–7 folds in *Slrup* mutants, and only 1.5–2 folds in *SlRUP-GFP* overexpression lines (Fig. 1F). HY5 and CHS1 play key roles in anthocyanin biosynthesis. Transcription of both *SlHY5* and *SlCHS1* was induced by UV-B much more pronounced in *Slrup* than in wildtype and *SlRUP-GFP* lines (Fig. 1G & H, Fig. S3). Intriguingly, the UV-B-induced expression of *SlHY5* and *SlCHS* was similar in wildtype and *SlRUP-GFP* overexpression lines. Based on those observations, we conclude that SlRUP is a negative regulator of UVR8-mediated UV-B photomorphogenesis.

We tested possible mechanism for SlRUP in UV-B signaling. SlUVR8 protein levels were similar in *Slrup* mutant and wildtype (Fig. S4), suggesting that SlRUP does not regulate SlUVR8 protein levels. In response to UV-B, UVR8 homodimers dissociate rapidly to monomers, which trigger UV-B signaling in plants (Rizzini et al., 2011). We tested whether SlRUP regulates the dimer/monomer conformational change of SlUVR8. Under white light, SlUVR8 was detected as homodimer and UV-B induced the dissociation of homodimer into monomer in wildtype, the *Slrup* mutant and the *SlRUP-GFP* overexpression line. In wildtype seedling, SlUVR8 showed dimer recovery initiated within 15 min, and completed within 2 h post UV-B exposure. The rate of SlUVR8 redimerization in the *Slrup* mutant was slow and monomer was detectable even 4 h post UV-B (Fig. 1I, Fig. S5A). SlUVR8 redimeration appears to be slightly faster in *SlRUP-GFP* overexpression line than wildtype post UV-B (Fig. 1I, Fig. S5B). Thus, we conclude that SlRUP accelerates redimeration of UV-B-activated SlUVR8 monomer.

To survive in nature, plants need to acquire mechanisms to cope with high dose UV-B stress. We previously showed that the acclimation to high dose UV-B stress was dependent on photoreceptor SlUVR8 (Liu et al., 2020b). We tested whether SlRUP participates in UV-B stress tolerance. Exposure of seedlings to stress UV-B (broadband) caused seriously stress response in wildtype, *SlRUP-GFP*, and *Sluvr8* mutant seedlings, whereas, *Slrup* mutant was obviously more resistant (Fig. S6). Consistent with the previous report, a beforehand exposure of seedlings to photomorphogenic UV-B (acclimated) for 2 d enhanced tolerance to broadband UV-B stress in wildtype, but not in *Sluvr8* mutant (Fig. S6) (Liu et al., 2020b). Interestingly, this acclimation effect was attenuated in *SlRUP-GFP* lines and enhanced in *Slrup* mutants (Fig. S6). This result demonstrated that SlRUP could negatively regulate UV-B acclimation and tolerance to UV-B stress of tomato seedlings.

## Discussion

In agricultural, tomato seedlings may face sudden UV-B exposure at seedling stage when transferred from UV-B-shielded green house to UV-B-rich open field. Thus, the understanding of UV-B responses of tomato seedlings is important for better agriculture practice. In this work, we characterized a key negative regulator of UV-B signaling pathway in tomato. *Slrup* mutants were hypersensitive to low fluence rate UV-B in the assays tested including hypocotyl elongation, UV-B target gene expression and anthocyanin accumulation. Through comparison of SlUVR8 photocycle in wildtype, *Slrup* mutant and *SlRUP* overexpression line, it is evident that SlRUP inhibits UV-B signaling via promoting the reversion of active SlUVR8 monomer to inactive homodimer. This mechanism is well conserved in Arabidopsis (Heijde and Ulm, 2013). Previous study showed that pigments and carotenoids are altered in *SlRUP*-RNAi tomato fruits (Liu et al., 2004). Further work is needed to investigate how SlRUP regulates tomato fruit metabolisms.

In a previous report, the *SlRUP*-RNAi line developed shorter hypocotyl and dark green leaves than wildtype in white light and dark (Liu et al., 2004). Thus, SlRUP is a negative regulator of photomorphogenesis not only in UV-B but also in white light. Intriguingly, overexpression of *SlRUP* also leads to shorter hypocotyls in white light. Thus, balanced expression of *RUP* is required for proper seedling photomorphogenesis. The mechanism for the function of SlRUP in white light awaits further investigation.

In summary, this study revealed that SlRUP is a key repressor of SlUVR8-mediated UV-B photomorphogenesis in tomato. SlRUP inhibits UV-B signaling via accelerating the conversion of active SlUVR8 monomer to inactive homodimer. In addition, SlRUP negatively regulates UV-B stress tolerance of tomato seedlings, which is important for agriculture practice.

### Supplementary Information

**Additional file 1.** Materials and Methods. Table S1: Primers used in this work

**Additional file 2 Fig. S1 Structural conservation of Tomato RUP**. (A) Amino acid sequences alignment of SlRUP, AtRUP1, and AtRUP2. (B) Schematic representation of the protein domain structures of SlRUP, AtRUP1 and AtRUP2. WD40 represents WD40-repeats (Trp-Asp) domain. **Fig. S2 Generation of** ***Slrup*** **mutants and** ***SlRUP-GFP*** **overexpression lines.** (A) Immunoblotting analysis with *SlRUP-GFP* overexpression lines. An anti-GFP antibody was used for immunoblotting. Ponceau Staining serves as loading control. (B) Schematic illustration of the two sgRNAs target sites on the *SlRUP* genomic sequence, blue box represents exon. Two sgRNAs targeting to the *SlRUP* coding sequence are in red font, and PAM (protospacer adjacent motif) in gray. (C) Sequence-based genotyping of CRISPR/Cas9-*SlRUP* homozygous mutant. The target sites are underlined and the PAM are highlighted in gray. The deletions are indicated by dashes. (D) and (E) Amino acid sequences alignment of *SlRUP* wildtype, *Slrup-CR8* (C) and *Slrup-CR22* (D) mutant. **Fig. S3 Transcript expression of** ***SlHY5*** **and** ***SlCHS1*** **in response to UV-B in** ***SlRUP*** **transgenic lines.** qRT-PCR analysis of *SlHY5* (A) and *SlCHS1* (B) in response to UV-B in wildtype (AC, *Ailsa Craig*), *Slrup* mutant, and *SlRUP-GFP* lines for different UV-B radiation time. Mean and SE of three biological samples are presented. **Fig. S4 SlUVR8 protein levels in AC and** ***Slrup*** **mutant in white light and white light with supplemental UV-B.** 6 d old tomato seedlings were either grown in white light (−UV) or white light supplemented with UV-B (+UV) for 2 h. An anti-SlUVR8 antibody was used for immunoblot analysis. Ponceau Staining serves as loading control. **Fig. S5 SlRUP promotes redimerization of SlUVR8.** 6 d old seedlings of AC (*Ailsa Craig*), *Slrup* mutant and *SlRUP* overexpression seedlings were irradiated with broadband UV-B for 0.5 h, followed by recovery in white light (WL) for different time. Anti-SlUVR8 antibody was used as the primary antibody for immunoblot analysis. Heat-denatured protein samples demonstrated total amounts of SlUVR8 protein. Ponceau Staining serves as loading control. **Fig. S6. SlRUP negatively regulates UV-B acclimation and tolerance.** Tomato seedlings were grown for 7 d under white light (20 μmol·m^− 2^·s^− 1^), then seedlings were treated with white light supplemented with photomorphogenic UV-B (Philips TL20W/01RS, 1.5 μmol·m^− 2^·s^− 1^) for 2 d (acclimated) or white light for 2 d (control and non-acclimated). Seedlings were then irradiated with broadband UV-B for 1 h (non-acclimated and acclimated), or subjected to a 1 h mock treatment (control) under white light. Treated seedlings were further grown for 4 d under white light without UV-B before being photographed. (A) Representative seedlings of AC (*Ailsa Craig*), *Slrup* mutants, and *SlRUP-GFP* lines recovery in white light without UV-B for 4 d. (B) Mean and SE of seedling fresh weight were shown (*n* = 9).

## Data Availability

The author responsible for distribution of materials integral to the findings presented in this article in accordance with the policy described in the Instructions for Authors in Molecular Horticulture is: ruohe.yin@sjtu.edu.cn
