# Supplementary material for: Tomato SlRUP is a negative regulator of UV-B photomorphogenesis
Source: Mol Hortic. 2021 Aug 27;1:8. doi: 10.1186/s43897-021-00010-z (PMC10514922; doi:10.1186/s43897-021-00010-z)
Supplement: Supplementary file 1 — Additional file 1. Materials and Methods. Table S1: Primers used in this work [file 43897_2021_10_MOESM1_ESM.docx]

**Materials and Methods**

**1. Plant materials, growth conditions and UV-B irradiation.**

The tomato mutants used in this work are in the cultivar *Ailsa Craig* (AC) background. The *Lecop1like* (*Slrup*) and *Slhy5* mutants were generated in this work via CRISPR/Cas9 gene editing approach, *Slhy5* mutant was generated in another work (Zhang et al in preparation). The *Sluvr8* mutant was described before (Liu, X. et al., 2020). Tomato seeds were surface-sterilized and sown on half-strength Murashige and Skoog basal salt medium (MS), germinated for 2 d in the dark, then seedlings were grown at 25 °C in a standard growth chamber under constant white light intensity (3.6 μmol m^-2^ s^-1^, measured by LI-250A Light Meter) for 4 d. For photomorphogenic UV-B treatment, tomato seedlings were grown as described above in 1/2 MS medium under white light supplemented with narrowband UV-B (Philips TL20W/01RS; 1.5 μmol·m^−2^·s^−1^). For SlUVR8 photocycle, 6 d old tomato seedlings grown under white light conditions were exposed to broadband UV-B (Philips TL40W/12RS; 12 μmol·m^−2^·s^−1^) for 0.5 h, followed by recovery in white light in the absence of UV-B for different periods as indicated.

**2. Quantitative Real-Time PCR**

Tomato total RNA was isolated with Plant RNeasy kit (OMEGA), and cDNA was synthesized with TransScript SuperMix (TransGen Biotech). Actin (*Solyc03g078400*) serves as the reference gene. PCR reactions were performed with Fast SYBR Green Master Mix (TransGen Biotech) on a CFX96 Real-time Fluorescence quantitative RT-PCR system (Bio-Rad). The expression of each gene at each time point after UV-B treatment was normalized against the expression in white light condition (UV-B 0 h). Primer pairs for *SlRUP*, *SlHY5*, *CHS1* and Actin are listed in Table S1. Three biological samples were analyzed for each time point in each experiment.

**3. Generation of SlRUP CRISPR/Cas9 mutant and SlRUP over-expression lines**

The CRISPR/Cas9 binary vector (pTX) was derived from pBin19, in which the target sequence was driven by the tomato *U6* promoter, and *Cas9* was driven by *CaMV35S*. The recombinant pTX vector was designed to produce mutation within the coding sequence of *SlRUP* using two sgRNAs alongside the *Cas9* gene. Agrobacterium tumefaciens mediated cotyledon explants transformation was used to produce tomato mutant, and AC (*Ailsa Craig*) was the background of transformation. Two CRISPR/Cas9 target sites were designed in the exon of *SlRUP*. For genotyping of every first generation (T_0_) transgenic lines, the DNA of T_0_ transgenic plants were employed as the template to amplify the targeted region fragments. Positive detection of each plant was conducted by PCR for the presence of *Cas9*, and then the CRISPR/Cas9 T-DNA positive lines were genotyped for mutations using the specific detect primers flanking the targeted sites. Then PCR products were sequenced using the specific primers to the sgRNA1 and sgRNA2 by TsingKe Biological Technology Company to detect the mutations.

For *SlRUP* over-expression generation, the *SlRUP-GFP* fusion sequence was recombined to pHellsgate8 vector, driven by *CaMV35S*. Agrobacterium tumefaciens mediated cotyledon explants transformation was used to produce the transgenic plants, and AC was the background of transformation. Immunoblot analysis was used to detect the *SlRUP-GFP* protein expression with anti-GFP antibody to select transgenic lines. The homozygous over-expression lines were obtained by selfing of the selected *SlRUP-GFP* expressing lines.

**4. Hypocotyl length measurement**

Tomato seedlings were grown as described above in 1/2 MS medium under white light (3.6 μmol m^-2^ s^-1^) or supplemented with narrowband UV-B (Philips TL20W/01RS; 1.5 μmol·m^−2^·s^−1^) after being germinated for 4 d. Around 30 seedlings were measured for each line, and hypocotyl length was quantified by ImageJ software (http://rsbweb.nih.gov/ij/). Significant difference between transgenic line and wildtype was detected by a one-way analysis of variance test.

**5. Anthocyanin extraction and quantification**

Anthocyanin extraction and quantification was performed according to the method published previously with modifications (Yin et al., 2012). Tomato seedlings were grown under white light or white light supplemented with low fluence rate UV-B as describe above for 4 d. 1 mL acidic methanol (1% HCl, w/v) was added to 200 mg seedling material. Anthocyanin was extracted at 4 °C for 1 h with moderate shaking. The homogenate was clarified by centrifugation,14000 rpm 5 min at room temperature. Anthocyanins were quantified based on the absorption at 530 nm and 657 nm by (A_530_-0.25 x A_657_)/M. A_530_ and A_657_ correspond to the absorptions at the indicated wavelengths, and M is the fresh weight of the samples used for extraction. Mean and SE of three independent biological replicates were obtained.

**6. Total protein extractions and immunoblots**

For SlUVR8 immunoblot, tomato total protein was extracted with buffer containing 50 mM Tris pH 7.6, 150 mM NaCl, 1 mM EDTA, 0.1% Tween 20, 1% protease inhibitor cocktail for plant extracts (Sigma) at 4 °C for 0.5 h with moderate shaking. After centrifugation at 4 °C, protein concentration of clear extracts was determined by Bradford method. Protein extracts were separated by electrophoresis in 10% SDS-PAGE and transferred to PVDF membranes according to the manufacturer’s instructions (Bio-Rad). Polyclonal antibodies anti-SlUVR8^415-429^ was used as primary antibodies and commercial anti-rabbit as secondary antibody. Signals were detected using the ECL reagent solution kit and a biostep Celvin S420 Chemiluminescence Imaging system.

**7. Plant growth conditions for UV-B acclimation and tolerance**

Tomato seeds were germinated for 2 d in the dark, then seedlings were grown at 25 °C in a standard growth chamber under constant white light intensity (20 μmol m^-2^ s^-1^, measured by LI-250A Light Meter) for 6 d. Then seedlings were treated with white light supplemented with photomorphogenic UV-B (Philips TL20W/01RS; 1.5 μmol·m^−2^·s^−1^) for 2 d (acclimated) or white light for 2 d (control and non-acclimated). The seedlings which were acclimated and non-acclimated were exposed to broadband UV-B (Philips TL40W/12RS; 21 μmol·m^−2^·s^−1^) for 1 h, followed by recovery in white light in the absence of UV-B for 4 d. The seedlings grown all through under white light as control.

**8. Accession numbers**

Tomato gene sequence data from this work can be retrieved from Sol Genomics Network (https://solgenomics.net/) under the following accession numbers: Solyc11g005190 (*SlRUP*), Solyc05g018615 (*SlUVR8*), and Solyc08g061130 (*SlHY5*).

**References**

**Liu, X., Zhang, Q., Yang, G., Zhang, C., Dong, H., Liu, Y., Yin, R., and Lin, L.** (2020). Pivotal roles of Tomato photoreceptor SlUVR8 in seedling development and UV-B stress tolerance. **Biochem Biophys Res Commun** 522**:** 177-183.

**Yin, R., Messner, B., Faus-Kessler, T., Hoffmann, T., Schwab, W., Hajirezaei, M.R., von Saint Paul, V., Heller, W., and Schaffner, A.R.** (2012). Feedback inhibition of the general phenylpropanoid and flavonol biosynthetic pathways upon a compromised flavonol-3-O-glycosylation. **J Exp Bot** 63**:** 2465-2478

**Table S1. Primers used in this work**

| Primer name | Primer sequence (5’- 3’) | Product length (bp) |
| --- | --- | --- |
| Slactin-q | Fw: AGGCAGGATTTGCTGGTGATGATGCT | 107 |
|  | Rv: ATACGCATCCTTCTGTCCCATTCCGA |  |
| SlHY5-q | Fw: AAGAGAGGAAGAAGCCCAGC | 89 |
|  | Rv: CTCTCCCTTGCTTGTTGTGC |  |
| SlCHS1-q | Fw: GCATACATGGCACCTTCCCT | 82 |
|  | Rv: TTTGGGCTGCCTCTTTTCCA |  |
| SlRUP-q | Fw: TATGAGGAAAATGCTTGACCCACT | 144 |
|  | Rv: ACCTTCTGGTCCTCCGCATTC |  |
| SlRUP-(C’) GFP | Fw: TTTGGAGAGGACACGCTCGAGATGACAGATATTTCATCAATTTTG | 1080 |
|  | Rv: AGTCAGATCTACCATCTCGAGTTTCCTCTTGCCATTAAAAACT |  |
| SlRUP-CRISPR | Fw: GAATCTAACAGTGTAGTTTG  GAAAAAGCAAGATGTGAATGTTTTAGAGCTAGAAATAGC | 700 |
|  | Rv: GCTATTTCTAGCTCTAAAAC  AGCTTCTGGTGGTATTGCTCAAACTACACTGTTAGATTC |  |
| SlRUP-CRdet | Fw: ATTCCTTTTTTGTCTCCTCCACT | 593 |
|  | Rv: AACCACTCCATCGTAATCACCC |  |
| CaMV35S-Fw | ACGCACAATCCCACTATCCTTC | -- |
| 35S-(C’) GFP-Rv | ATCACCTTCACCCTCTCCACTG | -- |
