# Supplementary material for: Tomato SlRUP is a negative regulator of UV-B photomorphogenesis
Source: Mol Hortic. 2021 Aug 27;1:8. doi: 10.1186/s43897-021-00010-z (PMC10514922; doi:10.1186/s43897-021-00010-z)
Supplement: Supplementary file 2 — Additional file 2 Fig. S1 Structural conservation of Tomato RUP. (A) Amino acid sequences alignment of SlRUP, AtRUP1, and AtRUP2. (B) Schematic representation of the protein domain structures of SlRUP, AtRUP1 and AtRUP2. WD40 represents WD40-repeats (Trp-Asp) domain. Fig. S2 Generation of Slrup mutants and SlRUP-GFP overexpression lines. (A) Immunoblotting analysis with SlRUP-GFP overexpression lines. An anti-GFP antibody was used for immunoblotting. Ponceau Staining serves as loading control. (B) Schematic illustration of the two sgRNAs target sites on the SlRUP genomic sequence, blue box represents exon. Two sgRNAs targeting to the SlRUP coding sequence are in red font, and PAM (protospacer adjacent motif) in gray. (C) Sequence-based genotyping of CRISPR/Cas9-SlRUP homozygous mutant. The target sites are underlined and the PAM are highlighted in gray. The deletions are indicated by dashes. (D) and (E) Amino acid sequences alignment of SlRUP wildtype, Slrup-CR8 (C) and Slrup-CR22 (D) mutant. Fig. S3 Transcript expression of SlHY5 and SlCHS1 in response to UV-B in SlRUP transgenic lines. qRT-PCR analysis of SlHY5 (A) and SlCHS1 (B) in response to UV-B in wildtype (AC, Ailsa Craig), Slrup mutant, and SlRUP-GFP lines for different UV-B radiation time. Mean and SE of three biological samples are presented. Fig. S4 SlUVR8 protein levels in AC and Slrup mutant in white light and white light with supplemental UV-B. 6 d old tomato seedlings were either grown in white light (−UV) or white light supplemented with UV-B (+UV) for 2 h. An anti-SlUVR8 antibody was used for immunoblot analysis. Ponceau Staining serves as loading control. Fig. S5 SlRUP promotes redimerization of SlUVR8. 6 d old seedlings of AC (Ailsa Craig), Slrup mutant and SlRUP overexpression seedlings were irradiated with broadband UV-B for 0.5 h, followed by recovery in white light (WL) for different time. Anti-SlUVR8 antibody was used as the primary antibody for immunoblot analysis. Heat-denatured protein sa [file 43897_2021_10_MOESM2_ESM.pptx]

## Slide 1
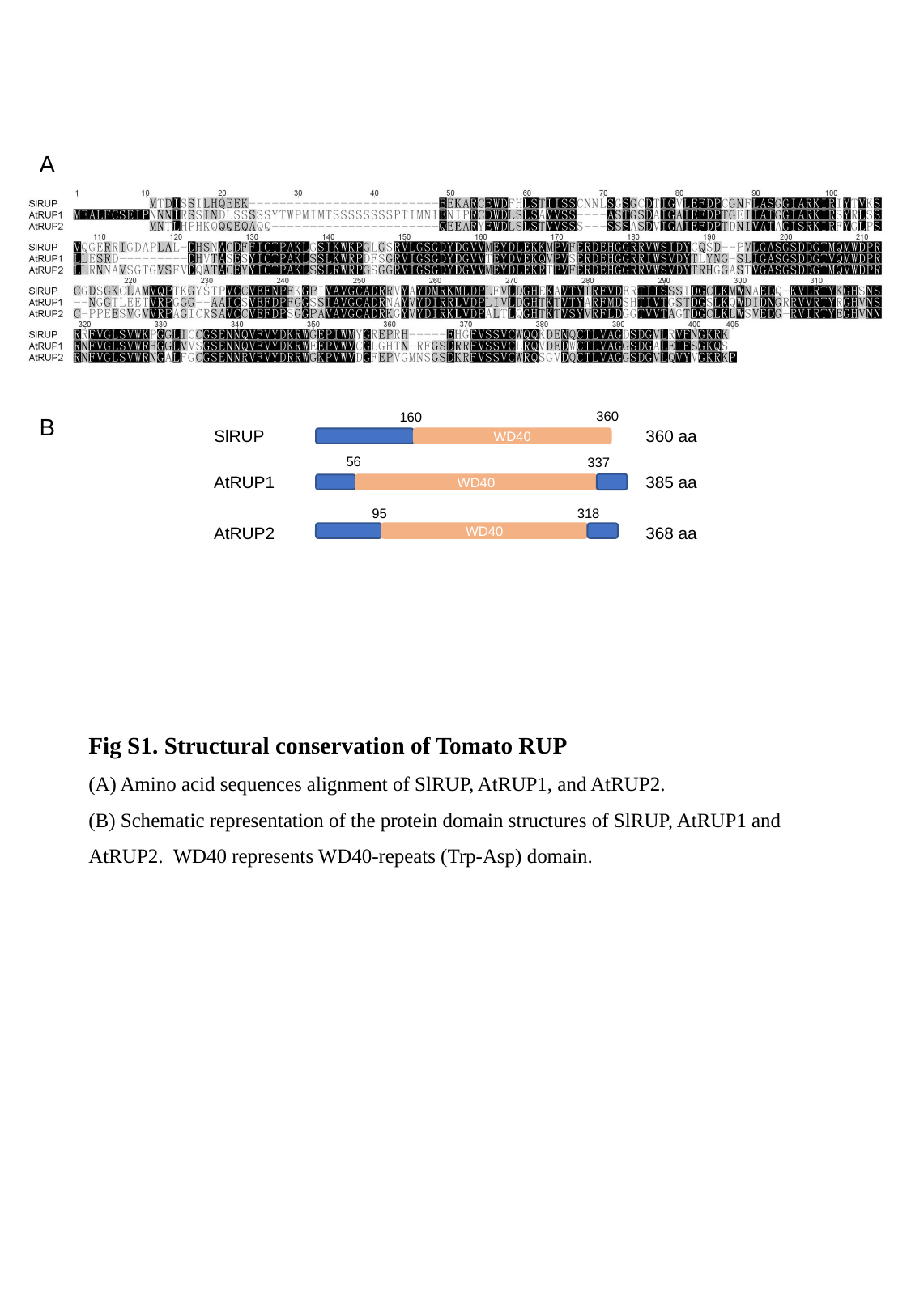

A
360
160
360 aa
SlRUP
WD40
56
337
AtRUP1
385 aa
WD40
318
95
AtRUP2
368 aa
WD40
B
Fig S1. Structural conservation of Tomato RUP
(A) Amino acid sequences alignment of SlRUP, AtRUP1, and AtRUP2.
(B) Schematic representation of the protein domain structures of SlRUP, AtRUP1 and AtRUP2. WD40 represents WD40-repeats (Trp-Asp) domain.

## Slide 2
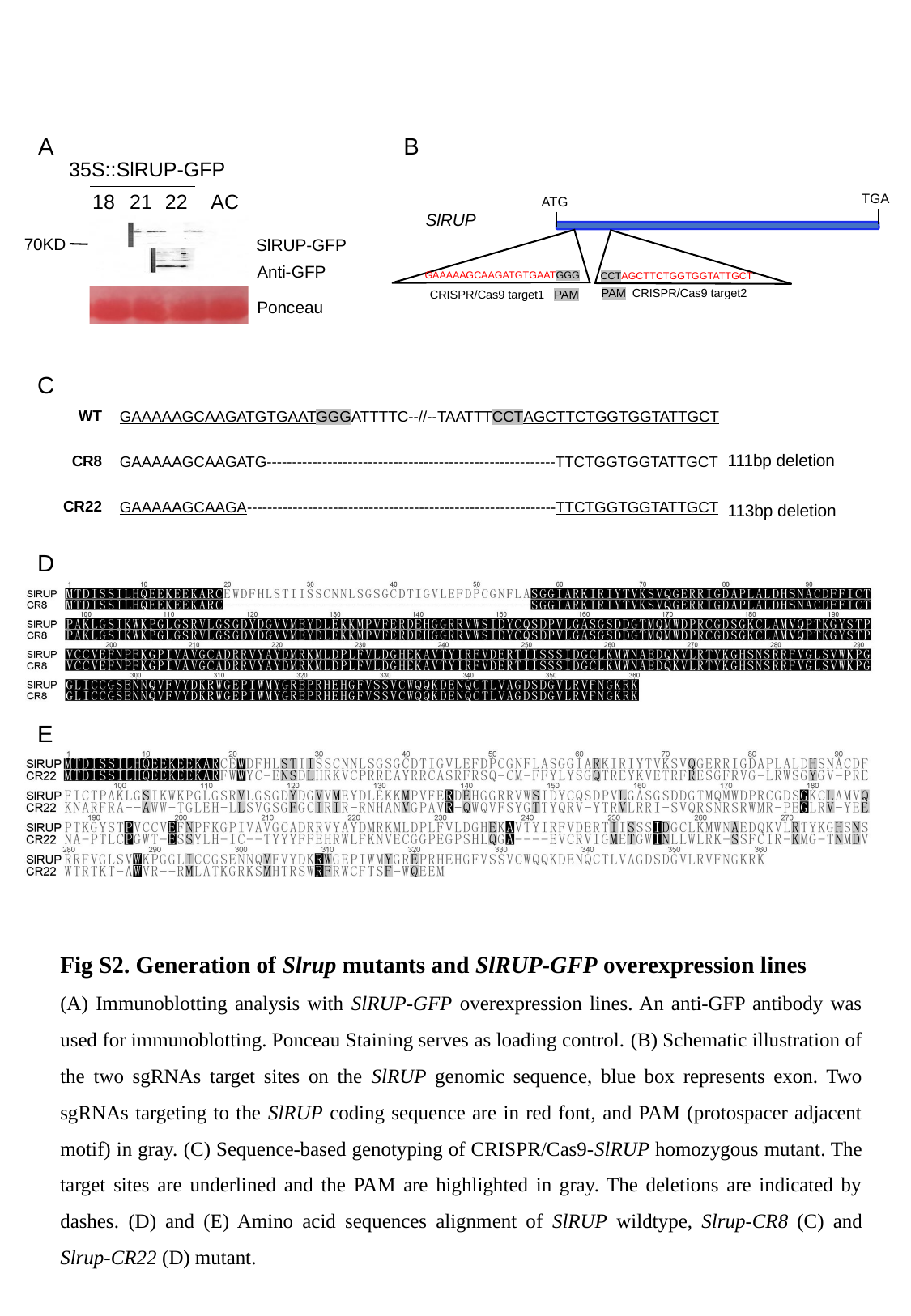

B
A
35S::SlRUP-GFP
22
AC
21
18
70KD
SlRUP-GFP
Anti-GFP
Ponceau
TGA
ATG
GAAAAAGCAAGATGTGAATGGG
CCTAGCTTCTGGTGGTATTGCT
PAM CRISPR/Cas9 target2
CRISPR/Cas9 target1 PAM
SlRUP
C
WT
CR8
CR22
GAAAAAGCAAGATGTGAATGGGATTTTC--//--TAATTTCCTAGCTTCTGGTGGTATTGCT
GAAAAAGCAAGATG---------------------------------------------------------TTCTGGTGGTATTGCT
GAAAAAGCAAGA-------------------------------------------------------------TTCTGGTGGTATTGCT
111bp deletion
113bp deletion
D
E
Fig S2. Generation of Slrup mutants and SlRUP-GFP overexpression lines
(A) Immunoblotting analysis with SlRUP-GFP overexpression lines. An anti-GFP antibody was used for immunoblotting. Ponceau Staining serves as loading control. (B) Schematic illustration of the two sgRNAs target sites on the SlRUP genomic sequence, blue box represents exon. Two sgRNAs targeting to the SlRUP coding sequence are in red font, and PAM (protospacer adjacent motif) in gray. (C) Sequence-based genotyping of CRISPR/Cas9-SlRUP homozygous mutant. The target sites are underlined and the PAM are highlighted in gray. The deletions are indicated by dashes. (D) and (E) Amino acid sequences alignment of SlRUP wildtype, Slrup-CR8 (C) and Slrup-CR22 (D) mutant.

## Slide 3
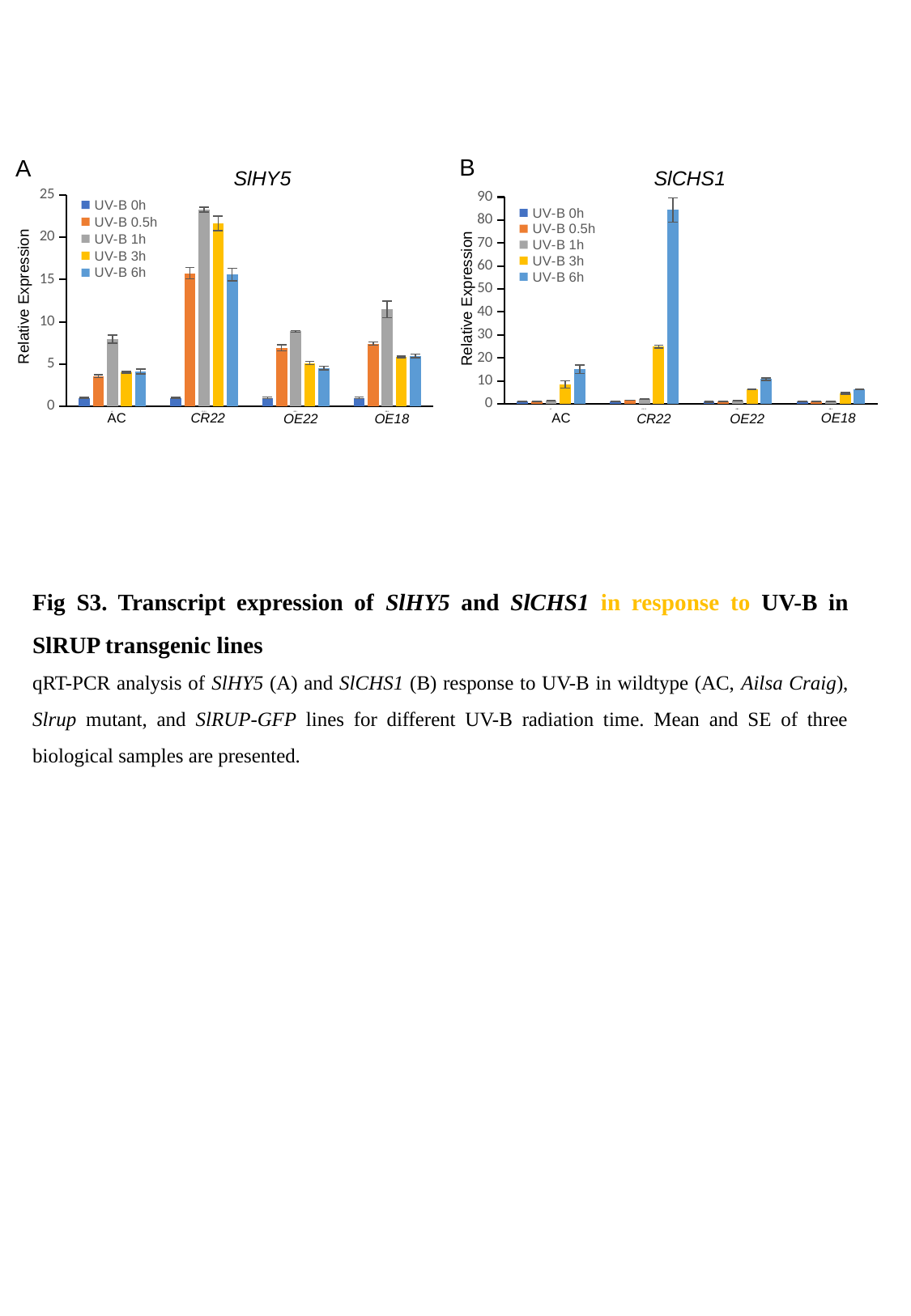

B
SlCHS1
### Chart
| Category | UV-B 0h | UV-B 0.5h | UV-B 1h | UV-B 3h | UV-B 6h |
|---|---|---|---|---|---|
| AC | 1.0 | 1.07056043863592 | 1.29053891955621 | 8.45522226008353 | 15.0068897060351 |
| CR22 | 1.0 | 1.63301611445355 | 2.259079316083 | 24.8883128603839 | 84.3887226800534 |
| OE22 | 1.0 | 1.09571812936033 | 1.40699672577725 | 6.34192026042098 | 10.7537935966709 |
| OE18 | 1.0 | 1.2149993390062 | 1.04808670328573 | 4.60286236698488 | 6.41890605505465 |Relative Expression
AC
OE18
CR22
OE22
A
SlHY5
### Chart
| Category | UV-B 0h | UV-B 0.5h | UV-B 1h | UV-B 3h | UV-B 6h |
|---|---|---|---|---|---|
| AC | 1.0 | 3.5624280820651 | 7.96659340276204 | 4.03124137518353 | 4.12250892456254 |
| CR22 | 1.0 | 15.7391377771374 | 23.2817120562781 | 21.6393014798009 | 15.5898546015884 |
| OE22 | 1.0 | 6.9108990879202 | 8.86503385980001 | 5.12171788622931 | 4.52570518695806 |
| OE18 | 1.0 | 7.41636106462521 | 11.4504995940484 | 5.83687432584475 | 5.94405778172878 |Relative Expression
AC
CR22
OE18
OE22
Fig S3. Transcript expression of SlHY5 and SlCHS1 in response to UV-B in SlRUP transgenic lines
qRT-PCR analysis of SlHY5 (A) and SlCHS1 (B) response to UV-B in wildtype (AC, Ailsa Craig), Slrup mutant, and SlRUP-GFP lines for different UV-B radiation time. Mean and SE of three biological samples are presented.

## Slide 4
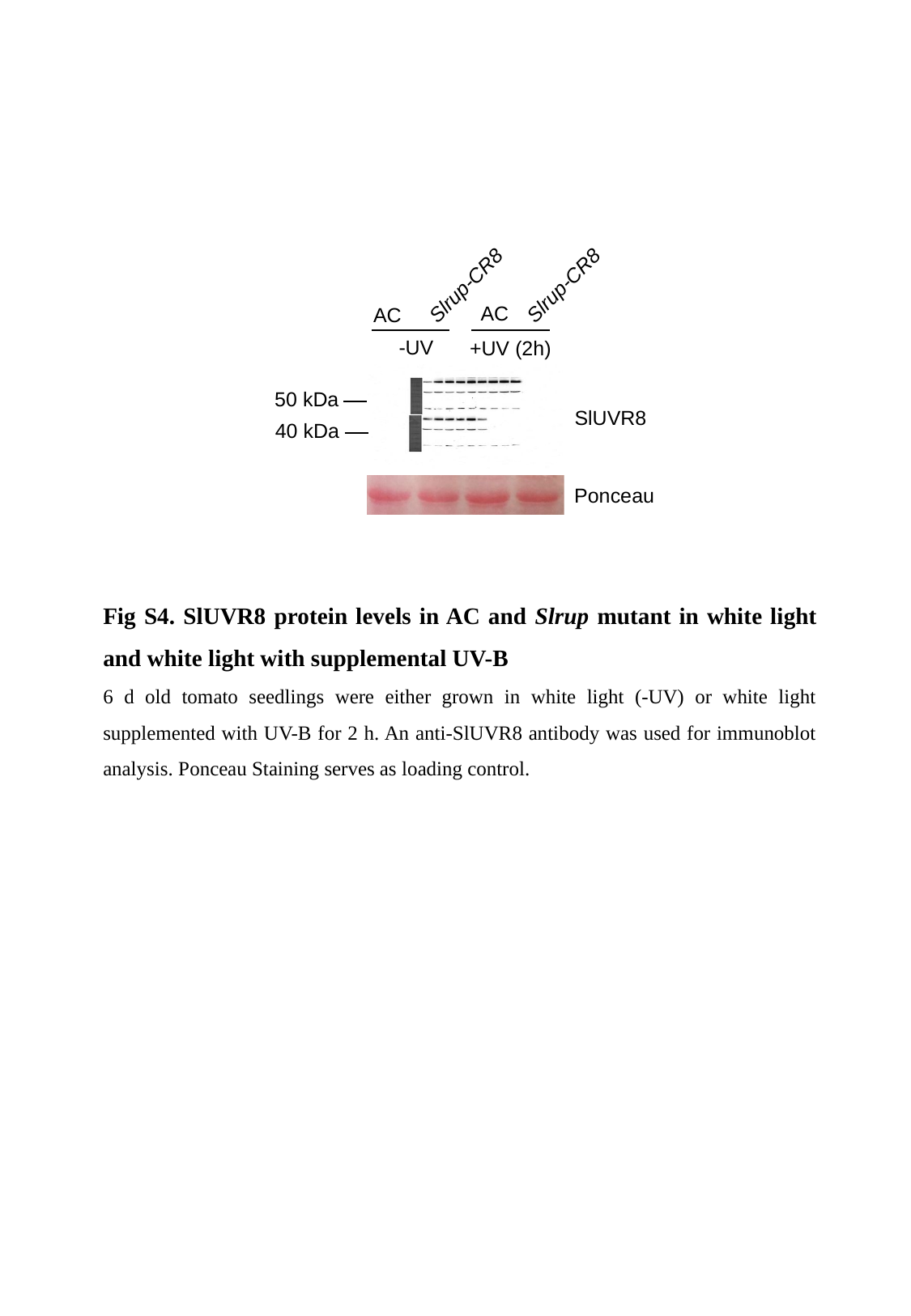

Slrup-CR8
Slrup-CR8
AC
AC
-UV
+UV (2h)
50 kDa
40 kDa
SlUVR8
Ponceau
Fig S4. SlUVR8 protein levels in AC and Slrup mutant in white light and white light with supplemental UV-B
6 d old tomato seedlings were either grown in white light (-UV) or white light supplemented with UV-B for 2 h. An anti-SlUVR8 antibody was used for immunoblot analysis. Ponceau Staining serves as loading control.

## Slide 5
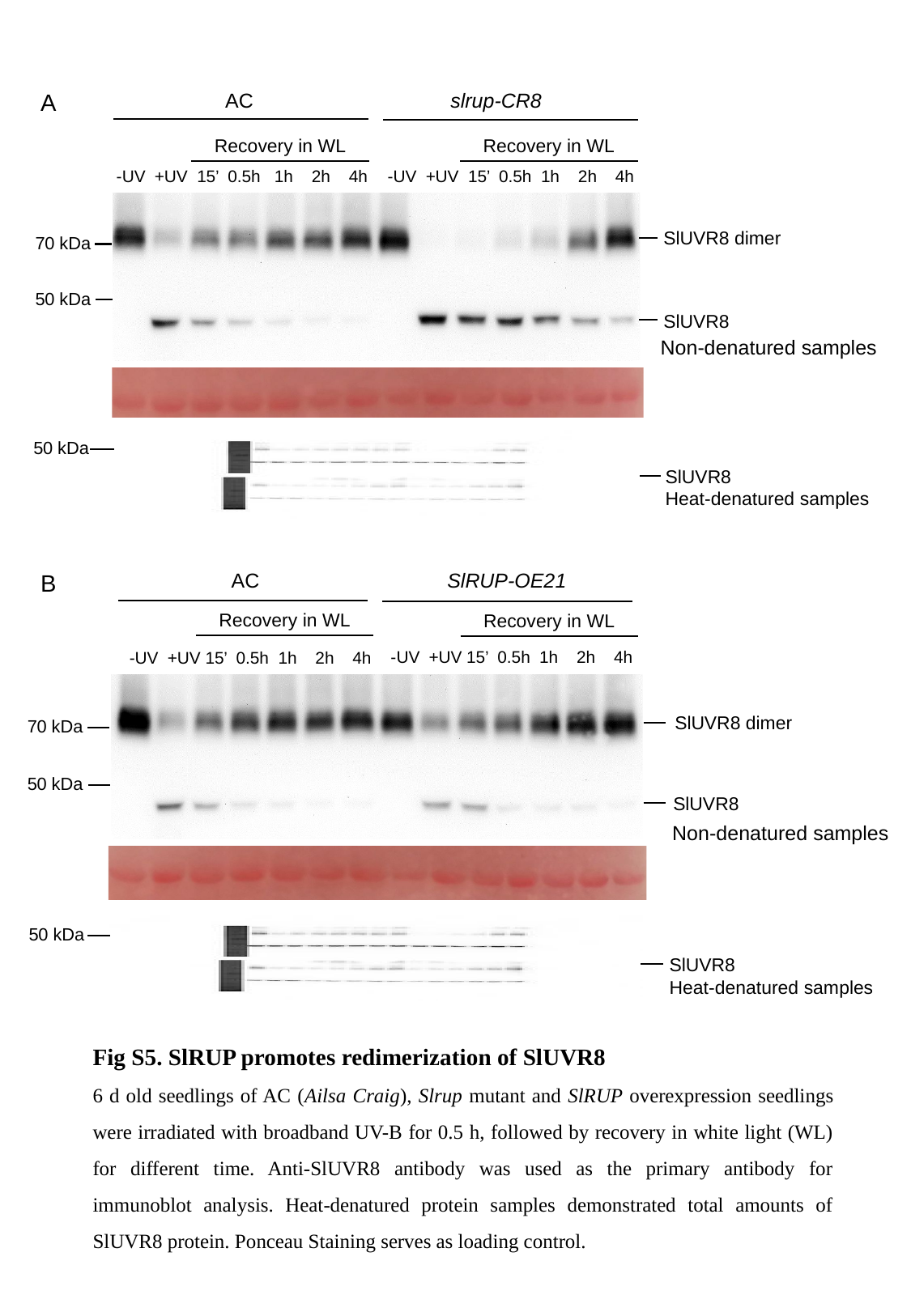

AC
slrup-CR8
-UV +UV 15’ 0.5h 1h 2h 4h
-UV +UV 15’ 0.5h 1h 2h 4h
70 kDa
50 kDa
SlUVR8 dimer
SlUVR8
50 kDa
SlUVR8
Heat-denatured samples
Recovery in WL
Recovery in WL
A
AC
SlRUP-OE21
-UV +UV 15’ 0.5h 1h 2h 4h
-UV +UV 15’ 0.5h 1h 2h 4h
70 kDa
50 kDa
SlUVR8 dimer
SlUVR8
Recovery in WL
Recovery in WL
50 kDa
SlUVR8
Heat-denatured samples
B
Non-denatured samples
Non-denatured samples
Fig S5. SlRUP promotes redimerization of SlUVR8
6 d old seedlings of AC (Ailsa Craig), Slrup mutant and SlRUP overexpression seedlings were irradiated with broadband UV-B for 0.5 h, followed by recovery in white light (WL) for different time. Anti-SlUVR8 antibody was used as the primary antibody for immunoblot analysis. Heat-denatured protein samples demonstrated total amounts of SlUVR8 protein. Ponceau Staining serves as loading control.

## Slide 6
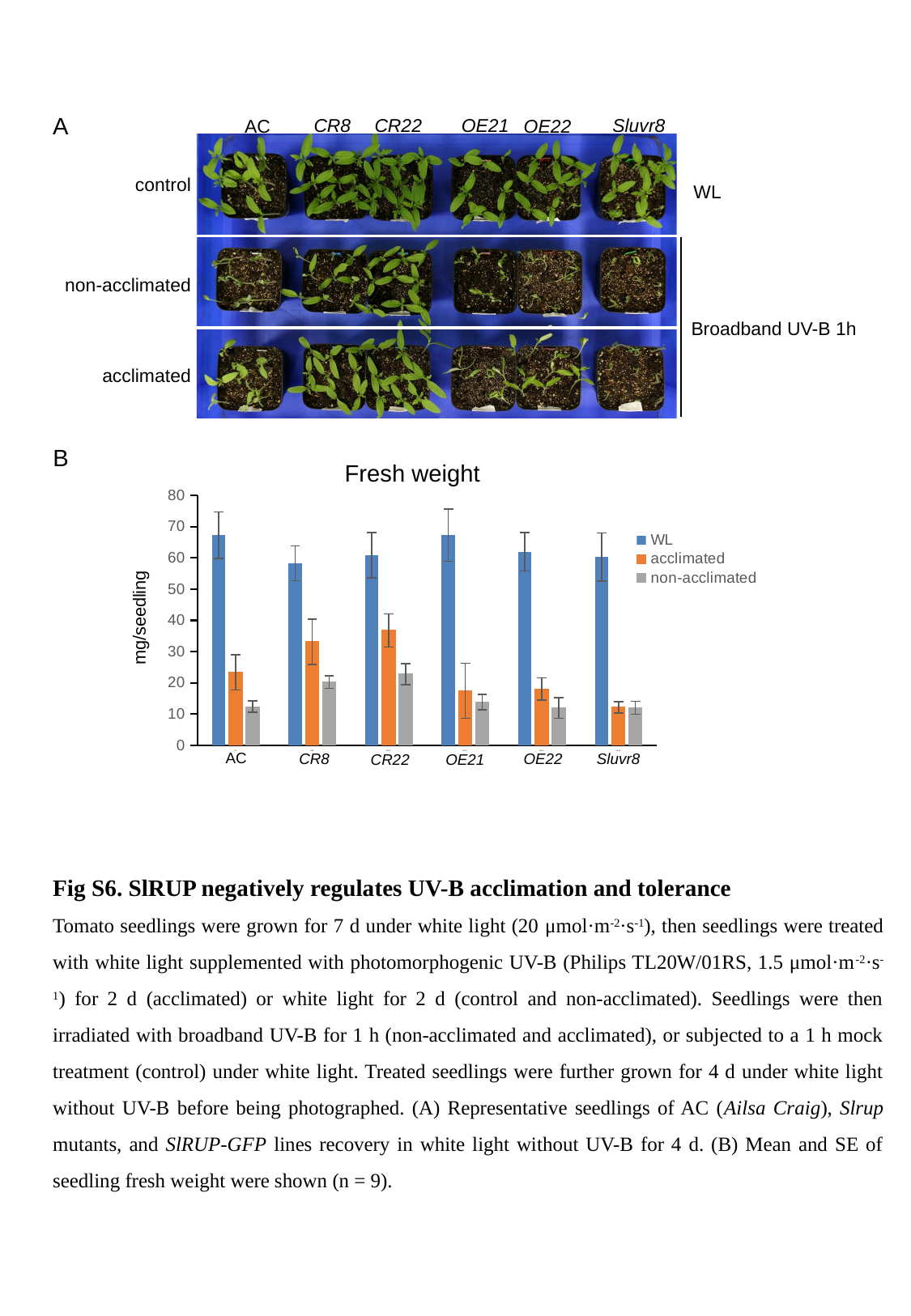

A
OE21
CR8
Sluvr8
AC
control
WL
non-acclimated
Broadband UV-B 1h
acclimated
CR22
OE22
B
Fresh weight
### Chart
| Category | WL | acclimated | non-acclimated |
|---|---|---|---|
| AC | 67.3333333333333 | 23.3333333333333 | 12.375 |
| CR8 | 58.2222222222222 | 33.1111111111111 | 20.25 |
| CR22 | 60.8888888888889 | 36.7777777777778 | 22.75 |
| OE21 | 67.25 | 17.4444444444444 | 13.7777777777778 |
| OE22 | 62.0 | 18.0 | 11.8888888888889 |
| Sluvr8 | 60.25 | 12.125 | 12.0 |mg/seedling
AC
CR8
OE22
Sluvr8
CR22
OE21
Fig S6. SlRUP negatively regulates UV-B acclimation and tolerance
Tomato seedlings were grown for 7 d under white light (20 μmol·m-2·s-1), then seedlings were treated with white light supplemented with photomorphogenic UV-B (Philips TL20W/01RS, 1.5 μmol·m-2·s-1) for 2 d (acclimated) or white light for 2 d (control and non-acclimated). Seedlings were then irradiated with broadband UV-B for 1 h (non-acclimated and acclimated), or subjected to a 1 h mock treatment (control) under white light. Treated seedlings were further grown for 4 d under white light without UV-B before being photographed. (A) Representative seedlings of AC (Ailsa Craig), Slrup mutants, and SlRUP-GFP lines recovery in white light without UV-B for 4 d. (B) Mean and SE of seedling fresh weight were shown (n = 9).
